# Civic Honors to a Medical Editor

**Published:** 1854-03

**Authors:** 


*Civic Honors to a Medical Editor.*—Jerome V. C. Smith, M. D., Editor of the Boston Medical and Surgical Journal, has been elected Mayor of the good city of Boston.

We do not know upon what ticket, whether whig, democrat, or independent, the Nestor of the medical corps editorial, has thus been elevated to the civic chair, but we are strongly inclined to side henceforth with that same party, whatever may be its cognomen. It is an evidence of a return to republican simplicity, of a due deference to medical honor and attainment; more than all, it is an appreciation of the medical editor.

From this time forward we set an increased value upon ourselves. As a man and practitioner we mean to be affable and condescending, but henceforth when we mount the tripod, a new dignity shall invest us, and an awful solemnity pervade our presence.

Times *are* improving. It is only a little reflected luster of honor that we borrow from Dr. Smith's elevation, but even that we appreciate. Now let Dr. J. V. C. Smith show the people of the "modern Athens," what a medical editor can do. Let him enlarge the hospitals, clean the streets, and instruct the functionaries who audit accounts, to pay the doctors' bills as presented. There is a wide field for reform, even in Boston, and Dr. Smith can add to his professional laurels, by making his official career one of sanitary usefulness.

We hear that Dr. Smith is a good business man as well as a good editor, and we do not doubt that his incumbency will be one of honor to himself, and usefulness to his city. H.

